# The Potential Role of the Extracellular Matrix Glycoprotein Reelin in Glioblastoma Biology

**DOI:** 10.3390/ph17030401

**Published:** 2024-03-21

**Authors:** Erika Ongemach, Daniela Zerrinius, Philipp Heimann, Christian Rainer Wirtz, Klaus-Michael Debatin, Mike-Andrew Westhoff, Aurelia Peraud

**Affiliations:** 1 Department of Pediatrics and Adolescent Medicine, Ulm University Hospital, 89075 Ulm, Germanyphilipp.heimann@uni-ulm.de (P.H.);; 2 Division Pediatric Neurosurgery, Department of Neurosurgery, Ulm University Hospital, 89081 Ulm, Germany; 3 Department of Neurosurgery, Ulm University Hospital, 89081 Ulm, Germany

**Keywords:** fibronectin, glioblastoma, motility, invasion, reelin, extracellular matrix

## Abstract

Glioblastoma, the most common and lethal primary adult brain tumor, cannot be successfully removed surgically due to its highly invasive nature. Therapeutically, approaches must be aimed at a systemic brain disease and not merely at a tumor located within the brain, unless a successful containment strategy can be found. Reelin, an extracellular matrix glycoprotein, plays an important role in neuronal migration and serves here as a natural stop signal. Interestingly, the expression of reelin is negatively associated with tumor grade and, within glioblastoma, correlates with increased overall survival. To further elucidate a potential biological reason for these findings, we looked at the cellular behavior of glioblastoma cell lines grown on a pure fibronectin matrix or a matrix with reelin inserts. While reelin had no significant effects on cellular metabolism, proliferation, or resistance to chemotherapeutic agents, it did significantly affect the cells’ interaction with fibronectin. Both matrix attachment and detachment were modulated by reelin, and thus, the invasion and motility of cells interacting with a reelin-containing matrix were altered. The data presented in this work strongly suggest that reelin might be a potential modulator of underlying molecular mechanisms that contribute to glioblastoma invasion.

## 1. Introduction

Glioblastoma (GB) is the most common primary brain tumor in adults and among the deadliest per se. It is not curable by surgery due to the extensive invasion of tumor cells into the healthy brain tissue [1], but is basically non-metastatic [2], therefore making the invasion localized, i.e., systemic for the whole brain, but without the involvement of other organs. GB is also a disease that is basically resistant to high doses of intensive radiotherapy and chemotherapy, leading to an average patient survival of well below 20 months [3].

There are several cellular aspects that make GB an extremely lethal and difficult-to-treat disease. Foremost among them, upon clinical presentation, these tumors are almost always already highly invasive and have infiltrated large areas of the brain, while, interestingly, they only rarely metastasize outside the neuroaxis [4]. This makes localized treatment, e.g., surgery, particularly ineffective [5], but even systemic chemotherapy or whole brain irradiation have so far proven to be insufficient at targeting cells not associated with the tumor bulk [4]. Both of these glioblastoma-specific aspects combined become apparent, for example, during radiotherapy, where those tumor cells not killed by the radiation exhibit increase in motility, i.e., become even more invasive (reviewed in [6]).

In recent years, we have developed the so-called Alcatraz Strategy [7], a complex combination therapy that aims at preventing glioblastoma cells from communicating via these contact points with their surrounding and, in essence, isolating them within the brain, thus reducing both invasion and apoptosis resistance [8,9,10]. Targeting the interaction of tumor cells with their microenvironment is a promising therapeutic approach, as it avoids inducing strong selective pressure on the genetically unstable tumor cell, mutational escape, and therapy resistance. However, the targets for such an intervention have to be selected carefully so as to prevent epithelial-to-mesenchymal transition (EMT), which can be induced by blocking cell–cell contacts and essentially leads to increased invasive behavior [11]. So far, blocking gap junctions in glioblastoma [8,10,12] has shown to be an extremely efficient approach that is currently being clinically evaluated. Furthermore, we could also prove that glioblastoma cells, when stressed by a hostile or non-permissive microenvironment, activate NF-κB, which leads to the production of a fibronectin matrix, indicating that invading glioblastoma cells create their own microenvironment in the brain [9]. Repurposing the anti-alcohol abuse drug Antabuse inhibits NF-κB activation and, thus, reduces the invasive potential of glioblastoma cells [9]. Importantly, both approaches, blocking gap junctions and inhibiting the NF-κB stress response, are likely to target different cellular subpopulations within the tumor and, thus, are ideal combination partners [10].

Reelin (RELN), an extracellular matrix glycoprotein, is known to play a major role in neuronal migration during the development of the central nervous system (cns), where it serves as a natural stop signal [13]. It inhibits signaling via integrins by directly disrupting the binding of α3β1 integrin to laminin and the binding of α5β1 integrin to fibronectin in a DAB1-dependent manner which leads to a migration stop of the respective neurons [14]. In recent years, it has been established that reelin could also play a decisive role in tumorigenesis inside and outside the central nervous system, as the expression of reelin is frequently altered in cancer. In glioblastoma, the expression of reelin is regularly downregulated by promoter methylation, which, in turn, is associated with a more aggressive phenotype of the tumor and leads to a poorer prognosis for the patient. In particular, the ability of the cells to migrate and invade into the surrounding brain tissue appears to be linked to the expression of reelin [15,16,17].

Here, we postulate that reelin, probably the 180 kDa isoform of reelin [13], might also be utilized in the Alcatraz Strategy, as it seems that the incorporation of reelin into a matrix can impair the interaction of glioblastoma cells with their microenvironment. This project is designed to investigate whether the natural properties of reelin can be harvested as part of an anti-invasive strategy that reduces the invasive potential of glioblastoma cells and turns the malignancy into a more localized and easier-to-treat disease.

## 2. Results

### 2.1. Reelin Expression Is Associated with Tumor Grade as Well as the Survival of Glioblastoma Patients

We initially hypothesized that, if reelin plays a contributing role in brain tumor biology, one would expect differing expression of this protein in healthy and malignant tissue. To test this, we accessed two publicly available data sets that look at RNA expression in the brain, made available through the REMBRANDT study and The Cancer Genome Atlas (TCGA). For further details, please see the corresponding section in Materials and Methods. Initially, we compared healthy tissue with increasing tumor grades, from I to IV, and could identify a continuous reduction in reelin expression correlating with the increasing tumor grade. Statistical analyses and the utilization of additional data sets are summarized in Table 1, Table 2, Table 3 and Table 4.

**Figure 1:**
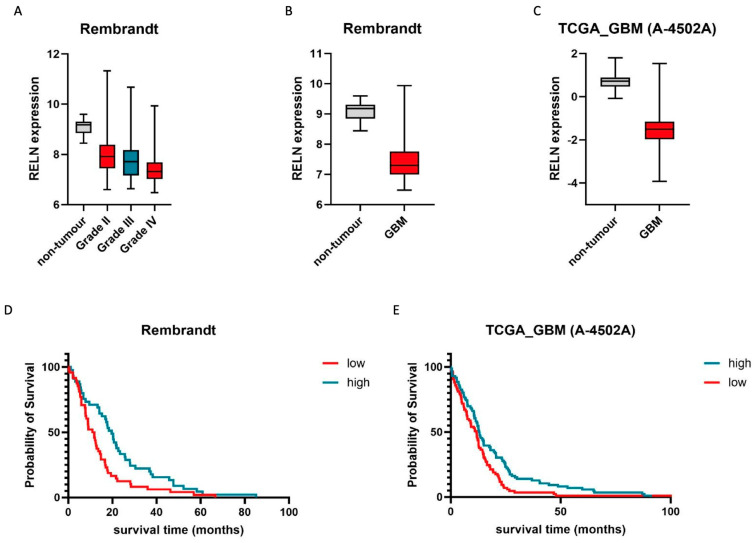
The presence of reelin correlates with patients’ survival and tumor grade. (**A**) Relative expression of RELN is inversely correlated with higher tumor grade. Non-tumor controls (n = 28) are indicated in grey, whilst grades II (n = 98), III (n = 85), and IV (n = 130) are indicated in red, blue, and red, respectively. Whiskers demark min. and max. values. THSD: Grade IV vs. I: 0.17 to −2.36, *p* = 0.11; grade IV vs. II: −0.31 to −0.71, *p* < 0.001; grade IV vs. III: −0.08 to −0.58, *p* < 0.01. (**B**,**C**) Glioblastoma (GB) samples express significantly lower amounts of RELN than healthy tissue. Non-tumor controls (B: n = 28, C: n = 10) are indicated in grey, whilst GB tissue ((**B**) n = 219, (**C**) n = 489) is indicated in red. Whiskers demark min. and max. values. THSD: B: −1.77 to −2.71, *p* < 0.001; C: −1.19 to 1.98, *p* < 0.001. (**D**,**E**) High expression of RELN correlates with longer patient survival. For KME, top (blue line, high) and bottom quartiles (red line, low) were chosen as cutoff points. In (**D**), Q1 and Q4 comprise n = 45 and n = 48 patients, whilst in (**E**), Q1 and Q4 comprise n = 86 and n = 85 patients (discrepancies in quartile size are due to missing survival data in some samples). Statistical significance (log rank): (D) *p* = 0.034; (E) *p* = 0.008. Representative Rembrandt (**A**,**B**,**D**) and TCGA (A 4505-A) (**C**,**E**) data sets were chosen for visualization.

As reelin is predominantly described, at least in the context of the brain, as a neuronal stop signal [13], we focused our analyses on glioblastoma, a grade IV astrocytoma, the most aggressive brain tumor in adults, which is characterized by its highly motile nature. Comparing reelin mRNA expression in healthy brain tissue and glioblastoma, a significant difference can be observed in three data sets (Table 1, Table 2, Table 3 and Table 4, examples in Figure 1B,C).

While these findings are indicative of a potential role for reelin in brain tumor biology, they lack clinical relevance per se. For example, an alternative explanation for the reduced presence of reelin messenger RNA in glioblastoma could be the high percentage of TAMs (tumor-associated macrophages) in glioblastoma, which have not been reported to express reelin so far. If the reduction in reelin expression in glioblastoma is of clinical relevance, this should be mirrored in relative patient survival. To this end, we utilized four independent data sets and could identify a positive correlation between reelin expression and overall patient survival in the context of glioblastoma (Figure 1D,E, Table 1, Table 2, Table 3 and Table 4). These analyses strongly suggest that either reelin expression is a potential biomarker for glioblastoma patient survival, independent of the protein’s actual function, or that reelin might have a therapeutically relevant effect on glioblastoma cell behavior. Therefore, we next concentrated on the three key features of glioblastoma that make it such a difficult-to-treat disease (1. a high proliferative index, 2. increased therapy resistance, and 3. a highly invasive nature) and investigated reelin’s potential contribution to these cellular features.

### 2.2. Reelin Mediates Neither an Increase in Cell Number Nor Cellular Survival

Although previous analyses by others [15,16,17] suggested that reelin has an effect on cellular behavior, only the purified, soluble form was used. As reelin is predominantly present as a protein inserted into a matrix, we designed our experimental setup to reflect this. Choosing fibronectin, a protein we had previously shown to be secreted by glioblastoma cells and utilized as a survival aid [9,10], as a basis for the matrix, we compared the behavior of cells interacting with fibronectin with or without reelin inserts. The concentration of reelin within the fibronectin matrix was chosen to reflect physiological conditions within the brain.

**Figure 2:**
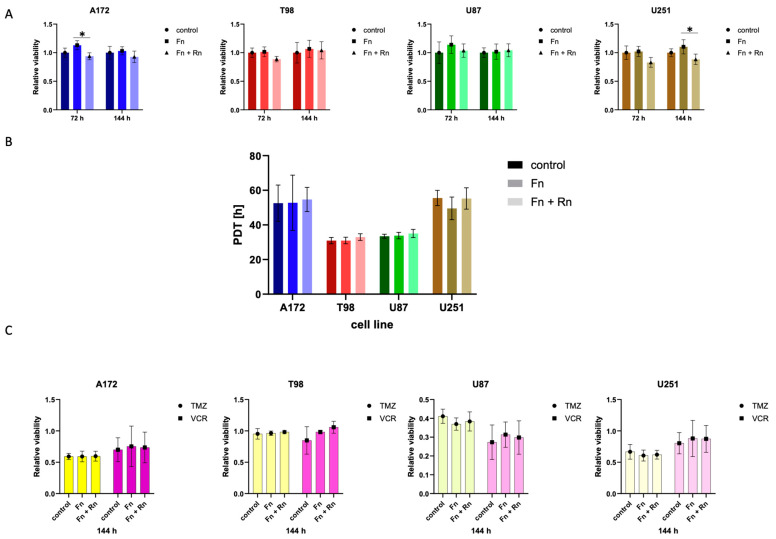
In all experiments performed (**A**–**C**), cells of four different glioblastoma cell lines were seeded at a density of 0.5 × 10^4^ cells/cm^2^ on either fibronectin or on a fibronectin matrix containing reelin inserts and incubated for 72 h or 144 h, respectively. As control, cells of each cell line were additionally seeded on uncoated wells. (**A**) The presence of RELN affects the metabolic activity of glioblastoma cells only temporarily. The metabolic activity of each cell population was normalized to the control. One-way ANOVA followed by Tukey’s multiple comparisons test were performed. The bars demonstrate the mean and the standard deviation of four independently performed experiments. * *<* 0.05. (**B**) RELN has no impact on the population doubling time of glioblastoma cells. The population doubling times were calculated as described above. One-way ANOVA followed by Tukey’s multiple comparisons test were performed. The bars demonstrate the mean and the standard deviation of three independently performed experiments. (**C**) The presence of RELN does not improve the therapeutic efficiency of commonly used chemotherapeutic agents. Cells were treated with 50 µM temozolomide (TMZ) or 0.5 nM vincristine (VCR) 24 h after seeding. To determine the therapeutic efficiency, the metabolic activity of each cell population was measured and normalized to cells that were solvent-treated. A two-way ANOVA was performed. The bars demonstrate the mean and the standard deviation of three independently performed experiments. The yellows bars demonstrate cells treated with TMZ, while the purple bars demonstrate cells treated with VCR. Grayscale was used to distinguish between the different cell lines.

First, we investigated whether the metabolic activity of the cells was affected by the presence of reelin. For this, we used the readout of relative cell population viability, as ascertained by the MTT assays after 72 and 144 h (Figure 2A). The presence of fibronectin had a transient effect on metabolic activity, which does not rise to statistical significance. This is in line with previous research, in which we showed that, upon seeding, glioblastoma cells can secrete their own fibronectin in an NF-κB-dependent manner [9]. The presence of reelin within the matrix seems to have a minor, transient negative effect at either 72 h for the A172 or 144 h for the U251 (Figure 2A), but no overall pattern was detectable. As the metabolic activity of cells should not be conflated with changes in cell number alone, we next calculated the population doubling time (PDT) of the four established cell lines used by counting cells (Figure 2B). No effect on PDT could be observed in cells seeded on fibronectin alone or on fibronectin and reelin when compared to (initially) plastic controls. Interestingly, the two cell lines with previously observed transient differences in metabolic activity (A172 and U251, Figure 2A) were the two slower proliferating cell lines in the set of four.

Based on these observations, it can be concluded that metabolic activity and proliferation are not aspects of cellular behavior strongly affected by the presence of reelin.

Second, we investigated whether response to therapy is affected by reelin. Hence, we chose two chemotherapeutic stimuli: temozolomide (TMZ) and vincristine (VCR). TMZ is an alkylating agent and the only chemotherapeutic that could show a survival benefit in patients with glioblastoma [18]. Vincristine is a vinca alkaloid that is commonly used in lower-grade glioma and experimentally in glioblastoma [19]. After incubating the various cell populations on different substrates for 144 h, viability was consistently reduced to about 50% after both treatments with TMZ and VCR (Figure 2C). However, neither the presence of fibronectin nor of fibronectin and reelin could improve the therapeutic efficiency of the two drugs employed (Figure 2C). The relative viability of the cell populations was comparable, regardless of the coating they were seeded on. No substantial differences were detectable in all four cell lines. Notably, T98, which is positive for O6-methylguanine–DNA methyltransferase expression (MGMT), appears highly resistant to DNA-damaging drugs when compared to the three other (MGMT-negative) cell lines.

In summary, it can be assumed that therapeutic efficiency is also not one of the main aspects affected by the presence of reelin. Third, we looked at the effect of a reelin-containing fibronectin matrix on the invasive behavior of glioblastoma cells. This aspect is of particular interest, as reelin is known to regulate neuronal migration during the development of the brain, and migration is an essential part of the invasive process.

### 2.3. Incorporated Reelin Acts as a Stop Signal and Slows down the Motility of Glioblastoma Cells

To ascertain the potential role of reelin in glioblastoma cell invasion, we broke down this highly complex process into several key aspects, namely cell attachment, cell detachment, cell transmigration through an existing matrix, and locomotive capacity.

**Figure 3:**
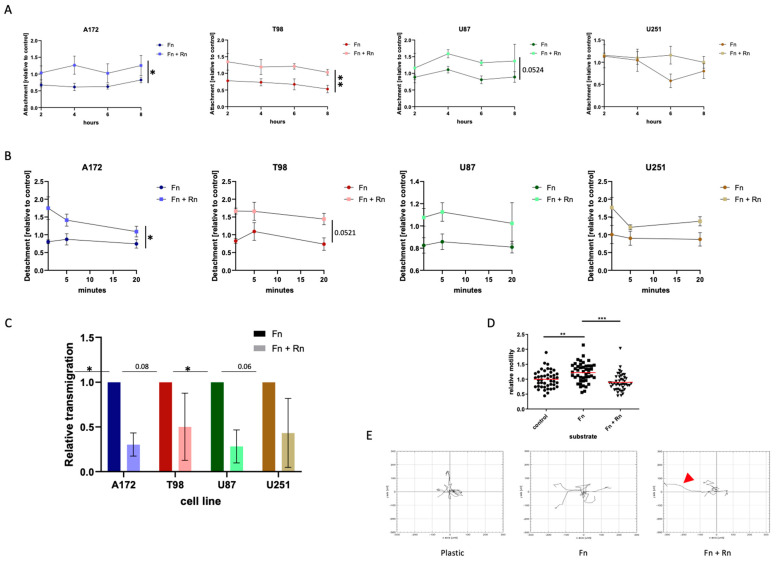
Effects of reelin on motility and invasion. (**A**,**B**) The presence of RELN can modulate both matrix attachment and detachment. (**A**) Attachment of the cells was measured 2, 4, 6 and 8 h after seeding. (**B**) To determine detachment, cells were seeded and allowed to settle down for 24 h. On the next day, cells were trypsinized and cell number was measured 1 min, 5 min, and 20 min after trypsin was added. In 3A,B multiple unpaired *t*-tests were performed, comparing the areas under the curve. The mean and the standard error of mean of three independently performed experiments are shown. * < 0.05; ** < 0.005. (**C**,**D**) Incorporated RELN can reduce the invasive capacity and the motility of glioblastoma cells. Glioblastoma cells were seeded at a density of 1 × 10^4^ cells/cm^2^ in Transwell chambers coated with fibronectin or fibronectin and reelin combined. As control (shown in (**D**)), cells were also seeded on uncoated Transwell chambers. Cells were allowed to transmigrate over a 24 h time span. After that, invasive cells were DAPI (4′,6-Diamidin-2-phenylindol)-stained and counted. (**D**) shows the effect of reelin incorporated into a fibronectin matrix on cell motility using the U87 cells, the glioblastoma cell line associated with the highest expression of reelin receptors. Three independently performed experiments are shown. In (**A**,**B**), the Mann–Whitney test is applied; in (**C**,**D**), a two-way ANOVA was performed. * < 0.05; ** < 0.005; *** < 0.001. (**E**) The presence of reelin can affect the migration route of glioblastoma cells. U87 cells were seeded on either fibronectin or on a fibronectin matrix containing reelin inserts. As controls, cells were additionally seeded on uncoated wells. The movement of five exemplary cells, randomly selected, per condition were tracked over an 8 h time span. The illustration shows the effect of reelin incorporated into a fibronectin matrix on cell migration routes using the U87 cells. The red arrowhead indicates a cell which detached and rolled, i.e., exhibited movement not controlled by traditional focal adhesion turnover.

Attachment: Freshly seeded cells were observed for a time span of eight hours. At two-hour intervals, the number of cells that had not attached yet was calculated as a fraction of the total cells seeded. As a fibronectin matrix has a known influence on attachment, only the comparison between fibronectin with fibronectin and reelin was made, and the development over time was based on the area under the curve, while cell culture-treated plastic was used as standard. Interestingly, for all four cell lines and all four time points, cells always attached less well in the presence of reelin, although statistical significance could only be achieved in two out of four cell lines (Figure 3A).

Detachment: The loss of adherence in the presence of trypsin, a digestive enzyme, was analyzed. As enzymatically induced detachment is a rapid process (also used in cell culture), a shorter time span of 20 min was selected, with measurements of detached cells after one, five, and twenty minutes. Conversely to the observations during the attachment experiments, the detachment of the cells was also affected by the presence of reelin, as cells detached faster and more easily from a matrix into which reelin was incorporated (Figure 3B). Again, this does not hold true for all cell lines in terms of overall statistical significance, but at all time points, a lower percentage of cells remained attached to a reelin-containing matrix.

Transmigration: To find out whether reelin incorporation can affect the ability of the cells to transmigrate through a matrix, in essence to invade the surrounding tissue, we performed Transwell assays, where we seeded a defined number of cells onto a porous membrane that was covered with either a fibronectin matrix or a fibronectin matrix that additionally contained reelin. Importantly, to transmigrate through the pores within the membrane, the cells would first need to degrade or otherwise manipulate the matrix. Figure 3C shows that the presence of reelin indeed affects the transmigration of cells when integrated into the fibronectin matrix. Despite high standard deviations in the T98 cells and the U251 cells, an impact of reelin regarding the ability to transmigrate was detectable in all four selected glioblastoma cell lines. However, it must be kept in mind that this experimental set-up is not independent of our previous findings, as a delay in adhesion after seeding might affect the cells’ ability to initiate the secretion of factors that disrupt the matrix, such as matrix metalloproteinases (MMPs), and thus initiate transmigration. All these experiments must be viewed as interconnected steps in the invasive cascade.

Locomotive capacity: Using the average velocity of a cell population, we investigated whether the presence of reelin influences the traction provided by a fibronectin matrix. Importantly, the experimental setup used here, the tracking of cells over time, does not identify the cause of potential differences, e.g., differences in number of focal adhesions or different kinetics of attachment and detachment, and therefore cannot be viewed as independent from the preceding sets of experiments.

Utilizing U87 cells, the most commonly used model cell line for glioblastoma, we compared average velocities of random movement on different substrates. Here, we could demonstrate that the velocity of cells is significantly increased when they are provided with a fibronectin matrix (Figure 3D). This increase in velocity is not only prevented by the addition of reelin but further reduced to levels below those of the plastic control.

In addition, we tracked five randomly selected U87 cells on the different substrates to demonstrate the effect of incorporated reelin on migration routes over a period of 8 h (Figure 3E). The red arrowhead indicates a cell on fibronectin and reelin that lost its traction, also indicating that the reduced velocity we observed is probably an underestimate, as rolling cells do not control the directionality of their movement and should not be assessed in the same way as motile cells.

In conclusion, this set of interconnected experiments shows clearly that the presence of reelin in a fibronectin matrix affects the interaction of glioblastoma cells with their surrounding, partially delaying their attachment, detachment, capacity to transmigrate through an existing matrix, and velocity during random movement. All these aspects investigated are closely associated with the invasive capacity of cells; therefore, we conclude that reelin, within a fibronectin matrix, has antiinvasive properties with respect to glioblastoma cells.

## 3. Discussion

In this work, we looked at the potential role of the glycoprotein reelin in the context of brain tumor therapy. A particular focus here was glioblastoma, a highly invasive tumor that, upon clinical presentation, will have invariably spread throughout the brain, and any treatment approach will, de facto, have to consider the treatment of the whole brain [10].

GB cells have long been shown to express a plethora of integrins, but research has been mostly focused on those containing the RGD (Arginyl-glycyl-aspartic acid)-binding motive, as these interact with matrix proteins and facilitate the activation of signaling hubs at the focal adhesion points [20,21]. Signaling via this integrin anchorage point is associated with both recurrence and drug-resistance in GB [22,23]; however, therapeutic exploitation of this feature, for example by cilengitide, an inhibitor of αvβ3 and αvβ5 [24], has not been translated into clinical success [25]. Recently, we showed that GB utilizes nodules of fibronectin present on their cell surface to alter their microenvironment to be more hospitable [9]. Upon cellular stress, NFkappa-B is activated and cleaves these fibronectin nodules in a metalloproteinase-dependent manner, which allows the establishment of a fibronectin-based matrix, with which the GB cells then interact in an integrin-dependent manner. The same metalloproteinase we identified in mediating this process, MMP-2 and MMP-9, are also involved in invasion via destruction of established matrices [26] and recurrence [27].

One of the signaling pathways often associated with focal adhesion signaling is the PI3-Kinase/Akt cascade, which also plays a pivotal role in GB biology, as its negative regulator, PTEN, is one of the few constantly altered targets in glioblastoma [21]. While PI3-Kinase/Akt is often associated with survival, it is much more complex than this [28]. Our own data suggests a rather minor role in mediating apoptosis resistance, but instead a large role in mediating motility in, at least, a subset of GB cells [29,30].

Reelin blocks signaling of the integrin subunit β1, particularly when it is paired with α3β1, often referred to as the laminin receptor [31,32]. The latter is the predominant integrin expressed by glioma cells, including the GB cell lines used in this study [33,34]. Interestingly, U87 cells are consistently referred to as high expressers [35].

In line with reelin’s commonly reported function as a neuronal stop signal [13], the analysis of publicly available data sets suggests that the expression of reelin is negatively associated with both tumorigenicity and tumor grade. Strikingly, within glioblastoma, a tumor that, despite intensive therapy, presents with an average patient survival of 18 months [3], reelin expression was clearly associated with increased survival. Importantly, these data sets do not allow speculation of the potential source of reelin, whether it is the particular brain region the tumor grows in, the non-tumorigenic cells that are associated with the tumor bulk, or, although counterintuitive, the tumor cells themselves. The latter cause would provide a new potential classification scheme and is currently the focus of further investigations, but clearly, further investigation of primary material, possibly on a single cell level, is needed.

Using four established cell lines, we next investigated the potential underlying effects reelin has on glioblastoma cell behavior that might account for the striking differences in patient survival. For this, we chose a fibronectin matrix as the fundament. Under these conditions, we could show that the presence of reelin within the fibronectin matrix had no significant effects on the overall cell fitness and population doubling time. Furthermore, under stress in the form of a chemotherapeutical attack, cells did not survive better in the absence of reelin. These data strongly support the notion that reelin is not a negative mediator of cell proliferation or survival. However, in line with its reported role as a stop signal, we showed that inserting reelin into the matrix reduced the strength of interaction between cell and substrate. Cells took longer to attach to and more readily detached from the matrix; they invaded the matrix less efficiently and exhibited reduced locomotive capacity. In summary, glioblastoma cells exhibited less invasive behavior in the presence of reelin. Importantly, as we did not use full-length reelin for our experiments but just the relevant peptide sequence that interacts with the known reelin receptors, it is unlikely that the observed effects are merely due to a mechanistic blockage due to the protein’s size.

It is tempting to speculate that, under the condition described, the effect of reelin is not mediated via downstream effectors, such as DAB1 (Disabled-1), but mainly functions by preventing engagement of the β1 integrin subunit with the matrix. This reduces the activation of the PI3-Kinase/Akt signaling cascade and reduces cellular motility, while only having minor effects on cell survival. The interaction between reelin and the PI3K/Akt pathway is complex and not associated with the negative effect postulated here, whereby reelin blocks the focal adhesion-mediated directional activation of PI3-Kinase signaling, but reelin, via DAB1, can also activate PI3-Kinase to control cortical development and to regulate dendritic growth [36]. In turn, PI3-Kinase has also been shown to enhance cell migration by explicitly downregulating reelin expression [37]. Clearly, more experiments are needed to elucidate the complex relationship between these two signaling modulators in GB cells.

While the therapeutic potential of reelin might not be apparent straightaway—after all, glioblastoma cells will have invaded the brain prior to clinical presentation—we feel that, therefore, prior to any potential medical invasion, utilizing part of its peptide sequence might be a valuable contribution to any multi-modular therapy.

After tumor removal, GB recurrence manifests within a distance of two to three centimeters from the resection cavity in more than 95% of cases [4], which suggests recruitment and re-population during the so-called Hydra Phenomenon [38] or induction by repair mechanisms similar to those seen after conventional chemo- and radiotherapy [39,40,41]. In addition, therapeutic interventions, such as radiotherapy or Bevacizumab treatment, have been linked to increased glioblastoma cell motility [42,43]. Therefore, carefully considered blockage of adhesion, possibly even only temporary, should be a potent addition to our (unfortunately, still rather restricted) arsenal against glioblastoma, mainly in extending the therapeutic window and reducing progressive neurological dysfunctions that are not necessarily associated with the tumor bulk [4]. Here, one could envision the utilization of TRUCKs (T cells redirected for universal cytokine-mediated killing) fourth-generation CAR (chimeric antigen receptor) T cells [44], to deliver a reelin peptide to the invading tumor cells and thus immobilize them.

While our study has clear limitations, such as the use of established cell lines and not primary patient material, it nevertheless clearly shows that further investigations into the potential use of reelin as a putative stop signal for glioblastoma invasion are warranted.

## 4. Materials and Methods

### 4.1. Publicly Available Patient Data

For exploratory data analysis, normalized expression data in combination with matched information on clinical outcomes collected by a total of six groups were sighted using GlioVis [45]. The respective data sets, namely Rembrandt, Gravendeel, Ivy Gap, Bao, CGGA, and TCGA (Agilent 4502-A microarray), were then imported and tested for a statistically significant effect of RELN expression on tumor grade, type, and overall survival [46,47,48,49]. To quantify potentially different RELN expression between healthy tissue and pathologically confirmed (IDH wild type) glioblastoma, as well as across tumor grades and types, Turkey’s honest significant difference (THSD) was calculated (Table 1, Table 2 and Table 4). A Kaplan–Meier estimator (KME) was used to model survival over time, for which significance was determined via log-rank test (Table 3). To reduce type 2 error, KME was performed at the median cutoff for top and bottom quartiles (high vs. low) and at the optimal cutoff calculated through maxrank statistics, as provided via GlioVis [50]. Unless stated otherwise, statistical analysis was conducted using GraphPad Prism 10.0.3 software.

### 4.2. Cells and Growth Conditions

The U87, T98, and A172 human glioblastoma cell lines were obtained from the American Type Culture Collection (Manassas, VA, USA), and the U251 human glioblastoma cell line was purchased from Sigma-Aldrich 01/2017 (St. Louis, MO, USA). All cells were stored in liquid nitrogen after initial expansion. New stocks were thawed after no more than thirty passages. All cells were maintained in Dulbecco’s Modified Eagle Medium (DMEM; Gibco, Life Technologies, Grand Island, NY, USA), supplemented with 10% fetal calf serum (FCS), 1 mmol/L glutamine, 1% penicillin/streptomycin, and 2.5% HEPES-Buffer (Biochrom AG, Berlin, Germany). Mycoplasma testing was carried out every six weeks.

### 4.3. Reagents

Fibronectin was purchased from Sigma-Aldrich (St. Louis, MO, USA), dissolved in sterile water, and stored at 2–8 °C. Partial human reelin (26.9 kDa) was purchased from Cusabio (Houston, TX, USA) and dissolved in sterile water. Stock was stored at −20 °C and working aliquots at 4 °C.

To investigate whether response to therapy is affected by reelin, two chemotherapeutic agents were chosen. Temozolomide (TMZ) is an alkylating agent and primarily a cytostasis-inducing drug [18]. TMZ was purchased from Sigma-Aldrich (Steinheim, Germany), dissolved in dimethyl sulfoxide (DMSO) (Sigma-Aldrich, Steinheim, Germany), and stored at −20 °C. Vincristine (VCR) is a vinca alkaloid and its primary mode of action is the induction of cell death [19]. VCR was obtained from the central pharmacy at Ulm University Medical Center (Ulm, Germany) and stored at 4 °C for a maximal duration of six weeks.

### 4.4. Coating

In all experiments performed, plates were left uncoated or coated with either fibronectin or the combination of fibronectin and reelin for 16 h, before cells were seeded. Final concentrations were set at 3.3 µg/cm^2^ for fibronectin and at 5 µg/mL for reelin. Uncoated wells served as control. Coated plates were incubated at room temperature overnight. Before seeding the cells, wells were washed with PBS (Gibco, Life Technologies, Grand Island, NY, USA) twice. The coating was reused up to three times.

### 4.5. Viability Assay (3-(4,5-Dimethylthiazol-2-yl)-2,5-Diphenyltetrazolium Bromide (MTT) Assay)

To examine cellular viability, 3-[4,5-Dimethythiazol-2-yl]-2,5-diphenyl tetrazolium bromide (MTT)-based assays were performed as previously described [51]. After coated plates were incubated for 16 h, cells were seeded at a density of 0.2 × 10^4^ cells/cm^2^ and incubated for 72 h and 144 h, respectively. Cell viability was quantified by measuring the optical density of each well with an automated microplate reader (ELx800; BioTek and Tecan infinite pro).

Additionally, we chose to employ metabolic viability as a readout to best depict the potential effects of TMZ and VCR. Therefore, we stimulated the cells 24 h after seeding with either 50 µM TMZ or 0.5 nM VCR, and cell viability was quantified 144 h after stimulation as described above.

### 4.6. Population Doubling Time as an Indicator of Proliferation

Plates were coated 16 h before cells were seeded, at a density of 0.2 × 10^4^ cells/cm^2^ in each well. Cells were allowed to settle and proliferate for 72 h and 144 h, respectively. At measuring time, cell number was determined using the CASY1 DT (Roche Diagnostics, Indianapolis, IN, USA), as previously described [29]. To calculate the population doubling time, the following website was used: https://www.doubling-time.com/compute.php (accessed on 24 September 2023).

### 4.7. Adhesion Assay

Cells were seeded at a density of 0.2 × 10^4^ cells/cm^2^ on coated plates and allowed to settle for 2 h, 4 h, 6 h, and 8 h, respectively. Once the determined time had passed, 100 µL of the cell solution was diluted in 10mL CASYton solution (OLS OMNI Life Sciences, Bremen, Germany) and cell number was measured using the CASY1 DT (Roche Diagnostics, Indianapolis, IN, USA). As the cells were not trypsinized before being added to the CASYton solution, the cells counted by the CASY1 DT correspond to the cells that had not been adherent to the coating so far.

### 4.8. Detachment Assay

Cells were seeded at a density of 0.2 × 10^4^ cells/cm^2^ on coated plates and allowed to settle overnight, to make sure most cells became adherent. Twenty-four hours after cells were seeded, trypsin/EDTA solution (Biochrom, Berlin, Germany) was added to the cells, and 100 µL of each well was taken and diluted in CASYton solution 1 min, 5 min, and 20 min after trypsin was added. The cells counted had already detached from the coating and could thereby be captured by using CASY1 DT.

### 4.9. Invasion Assay (Transwell Assay)

Cellular invasion was determined 24 h after cells were seeded at a density of 1 × 10^4^ cells/cm^2^, either on a coated or uncoated membrane in an 8 µm pore Transwell insert (Corning Inc., Corning, NY, USA). Medium containing 20% FCS (Biochrom, Berlin, Germany) served as a chemoattractant, while cells were seeded in medium without FCS. Membranes were cleaned and cut out of the inserts. Cells were DAPI-stained (Roche Diagnostics, Indianapolis, IN, USA) and counted using fluorescence microscopy (Olympus, Shinjuku, Prefecture Tokyo, Japan).

### 4.10. Velocity Assay

Random movement of cells was analyzed using time-lapse photography, by taking a picture every 20 min over a time course of 24 h. Relative speed of cells was quantified using ImageJ software 1.54g (Rasband, W.S., ImageJ, U.S. National Institutes of Health, Bethesda, MD, USA, 1997–2011). To track the movement of the cells, the MTrackJ Plugin was used. For each coating, five cells were selected and tracked over a time span of 8 h. The obtained data was imported in a Chemotaxis tool within ImageJ to illustrate the migration routes.

## Figures and Tables

**Table 1:** Reelin expression in patients. Differential expression by tumor grade.

	Grade IV vs. I		Grade IV vs. II		Grade IV vs. III		Grade II vs. I		Grade III vs. I		Grade III vs. II	
	THSD	*p*	THSD	*p*	THSD	*p*	THSD	*p*	THSD	*p*	THSD	*p*
TCGA (Agilent)	n.a.	n.a.	n.a.	n.a.	n.a.	n.a.	n.a.	n.a.	n.a.	n.a.	n.a.	n.a.
CGGA	n.a.	n.a.	−0.86, −1.13 to −0.60	<0.001	−0.47, −0.75 to −0.20	<0.001	n.a.	n.a.	n.a.	n.a.	−0.39, −0.66 to −0.12	<0.01
Rembrandt	−1.10. −2.36 to 0.17	0.11	−0.55, −0.79 to −0.31	<0.001	−0.33, −0.58 to −0.08	<0.01	−0.55, −1.81 to 0.72	0.68	−0.76, −2.03 to 0.50	0.40	−0.22, −0.48 to 0.05	0.15
Gravendeel	−1.07, −2.12 to 0.02	0.04	−1.10. −1.73 to −0.46	<0.001	−0.52, −0.91 to −0.13	<0.01	0.03, 1.15 to 1.21	1	−0.55, −1.62 to 0.51	0.54	−0.58, −1.25 to 0.09	0.11
Bao	n.a.	n.a.	n.a.	n.a.	n.a.	n.a.	n.a.	n.a.	n.a.	n.a.	n.a.	n.a.
Ivy GAP	n.a.	n.a.	n.a.	n.a.	n.a.	n.a.	n.a.	n.a.	n.a.	n.a.	n.a.	n.a.

THSD: Turkey’s honest significant difference, *p*: *p* value, TCGA: The Cancer Genome Atlas Program, CGGA: Chinese Glioma Genome Atlas, n.a.: no corresponding data available, Ivy GAP: Ivy Glioblastoma Atlas Project.

**Table 2:** Reelin expression in patients. Glioblastoma samples vs. healthy control.

	GB Samples vs. Healthy Control	
	THSD	*p*
TCGA (Agilent)	−2.24; −2.71 to −1.77	<0.001
CGGA	n.a.	n.a.
Rembrandt	−1.59; −1.98 to −1.19	<0.001
Gravendeel	−2.77; −3.93 to −1.61	<0.001
Bao	n.a.	n.a.
Ivy GAP	n.a.	n.a.

THSD: Turkey’s honest significant difference, *p*: *p* value, n.a.: no corresponding data available.

**Table 3:** Reelin expression in patients. Survival analysis.

	Median		High vs. Low (Q1 vs. Q4)		Optimal Cutoff	
	HR	*p*	HR	*p*	HR	*p*
TCGA (Agilent) *	1.43; 1.13 to 1.81	0.003	1.4; 1.03 to 2	0.034	1.45; 1.15 to 1.84	0.002
CGGA *	0.98; 0.71 to 1.34	0.893	1.03; 0.66 to 1.62	0.881	1.8; 1.01 to 3.19	0.042
Rembrandt **	1.63; 1.2 to 2.22	0.002	1.77; 1.16 to 2.69	0.008	1.8; 1.31 to 2.47	<0.001
Gravendeel *	2.07; 1.4 to 3.05	<0.001	2.91; 1.63 to 5.21	<0.001	2.54; 1.58 to 4.08	<0.001
Bao	n.a.	n.a.	n.a.	n.a.	n.a.	n.a.
Ivy GAP	n.a.	n.a.	n.a.	n.a.	n.a.	n.a.

HR: hazard ratio, *p*: *p* value (log-rank), n.a.: no corresponding data available * IDH (isocitrate dehydrogenase) wild-type ** IDH statuses unresolved.

**Table 4:** Reelin expression in patients. Differential expression by tumor type.

	Glioblastoma						Astrocytoma				Oligodendroglioma	
	Astrocytoma		Oligodendroglioma		Mixed Glioma		Oligodendroglioma		Mixed Glioma		Mixed Glioma	
	THSD	*p*	THSD	*p*	THSD	*p*	THSD	*p*	THSD	*p*	THSD	*p*
TCGA (Agilent)	n.a.	n.a.	n.a.	n.a.	n.a.	n.a.	n.a.	n.a.	n.a.	n.a.	n.a.	n.a.
CGGA *	−0.99, −1.38 to −0.61	<0.001	−0.73, −1.16 to −0.29	<0.001	n.a.	n.a.	0.27, −0.21 to 0.75	0.65	n.a.	n.a.	n.a.	n.a.
Rembrandt	−0.45, −0.66 to −0.23	<0.001	−0.42, −0.69 to −0.14	<0.001	−0.15, −0.76 to 0.47	0.98	0.03, −0.26 to 0.32	1	0.30, −0.32 to 0.92	0.74	0.27, −0.37 to 0.92	0.83
Gravendeel **	−0.78, −1.43 to −0.13	0.01	−0.66, −1.17 to −0.15	<0.01	−0.48, −1.14 to 0.17	0.29	0.12, −0.62 to 0.86	1	0.30, −0.55 to 1.14	0.92	0.18, −0.57 to 0.93	0.98
Bao ***	−1.51, −2.37 to −0.65	<0.001	−1.70, −2.78 to −0.62	<0.001	n.a.	n.a.	−0.20, −1.34 to 0.95	0.97	n.a.	n.a.	n.a.	n.a.
Ivy GAP	n.a.	n.a.	n.a.	n.a.	n.a.	n.a.	n.a.	n.a.	n.a.	n.a.	n.a.	n.a.

THSD: Turkey’s honest significant difference, *p*: *p* value, n.a.: no corresponding data available * authors additionally differentiate anaplastic astrocytoma, anaplastic oligodendroglioma, oligoastrocytoma, and anaplastic oligoastrocytoma ** authors additionally differentiate pilocytic astrocytoma *** authors additionally differentiate oligoastrocytoma.

## Data Availability

The results published here are, in part, based upon data generated by the TCGA Research Network: https://www.cancer.gov/tcga (accessed on 14 March 2024).
